# Reviewers Acknowledgement for 2020

**DOI:** 10.31372/20200504.1121

**Published:** 2021

The Editor-in-Chief of *Asian/Pacific Island Nursing Journal* would like to thank the following people who served as peer-reviewers for the journal from January 2020 through December 2020. Without your support the journal could not exist.

Kamomilani Anduha Wong

Richard Arakaki

Umanthesani Balakrishnan

Ruel Billones

Kathryn Braun

Mirella Brooks

Ha Do Byon

Rose Constantino

Chad Cross

Alona Dalusung-Angosta

Kathryn Daub

Felicitas dela Cruz

Gabriel Garcia

Artemis Gonzales

Naoko Hikita

Eun-Ok Im

Kamalnand Singh

Ira Kantrowizt Gordon

Merle Kataoka-Yahiro

Carina Katigbak

Masashi Kawano

May Kealoha

Sera Kim Sunada

Pauline Lasquete

Heeyoung Lee

Wen-Wen Li

Nada Lukkahatai

Miguel Luzviminda Banez

Angelina Nguyen

Connie Nguyen-Truong

Donna Marie Malakiko

Mary Frances Oneha

Misty Pacheco

Andrew Reyes

Karol Richardson

Reiko Sakashita

Leorey Saligan

Takuichi Sato

Hiroaki Satoh

Reimund Serafica

Chiraporn Tachaudomdach

Hatsumi Tanigiuchi

Lillian Tom-Orme

Jenny Tsai

Johnny Yao

Jenny Zhong

